# Specific frontal neural dynamics contribute to decisions to check

**DOI:** 10.1038/ncomms11990

**Published:** 2016-06-20

**Authors:** Frederic M. Stoll, Vincent Fontanier, Emmanuel Procyk

**Affiliations:** 1 Univ Lyon, Université Lyon 1, Inserm, Stem Cell and Brain Research Institute U1208, 18 avenue du Doyen Lepine, 69500 Bron, France

## Abstract

Curiosity and information seeking potently shapes our behaviour and are thought to rely on the frontal cortex. Yet, the frontal regions and neural dynamics that control the drive to check for information remain unknown. Here we trained monkeys in a task where they had the opportunity to gain information about the potential delivery of a large bonus reward or continue with a default instructed decision task. Single-unit recordings in behaving monkeys reveal that decisions to check for additional information first engage midcingulate cortex and then lateral prefrontal cortex. The opposite is true for instructed decisions. Importantly, deciding to check engages neurons also involved in performance monitoring. Further, specific midcingulate activity could be discerned several trials before the monkeys actually choose to check the environment. Our data show that deciding to seek information on the current state of the environment is characterized by specific dynamics of neural activity within the prefrontal cortex.

## Abstract (web summary)

Information seeking is thought to rely on the brain's frontal cortex but which regions specifically control this drive remains unknown. Here the authors show that monkeys deciding to seek information on the current state of the environment showed specific neural dynamics in the lateral prefrontal cortex and midcingulate cortex.

## Graphical abstract

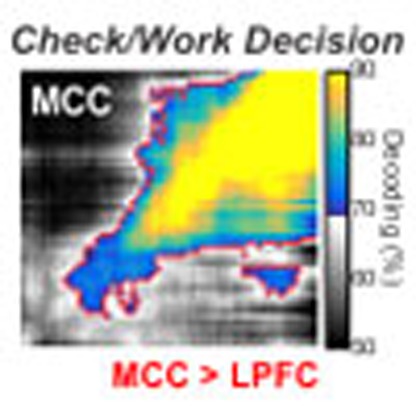

Beyond simple reactions to mistakes or successes, animals and, in particular, primates are capable of improving decision efficiency and reduce uncertainty by collecting additional information^1^. Foraging, for instance, is a highly adaptive activity that entails constant exploration for specific information and reactivity to sudden threatening cues. In more complex settings, verification of one's own performance or task completion represents another mode of information seeking that serves to increase the efficiency of behaviour. One critical question is how decisions to seek or check for information are produced in the brain.

The primate global cortical network contains a dense core of highly interconnected areas^2^, which covers a set of regions co-activated during adaptive decision-making^3^,^4^. Empirical descriptions complement theoretical accounts suggesting that parts of the dense core, especially the lateral prefrontal cortex (LPFC) and midcingulate cortex (MCC), contribute to different components of free, exploratory and adaptive decisions, especially in the context of adaptive responses to unexpected feedback^5^,^6^,^7^. However, both the experimental evidence and theory provide conflicting information on the synergy between MCC and LPFC. How and on the basis of what information they participate together in complex decisions remain unclear^8^,^9^,^10^,^11^. For example, MCC activation was shown to reflect the potential value, and thus the incentive, of foraging^12^. Yet, activation variations could also integrate multiple signals, including aspects of the difficulty of deciding and evaluating the need to shift from the default option^10^,^13^.

Decision-making is dynamically encoded in the frontal cortex^14^. Tonic and dynamical activity in LPFC contributes to decision-making based on active memory of external cues^15^. MCC neural activity reflects reinforcement-related information cumulated through trial and error, and maintained across trials, contributing to value-based decision-making^11^,^16^,^17^. However, decisions to check or seek for information are qualitatively different from decisions driven by environmental cues and hence might exhibit a specific frontal dynamic. A critical question thus concerns the specific and relative contributions of MCC and LPFC dynamics to information seeking and the mechanisms by which such behaviour is triggered.

In this context, our study builds on work linking MCC to foraging patches of rewarded options^12^,^18^, which might also link to the putative role of this area in obsessive compulsions marked by exaggerated checking^19^,^20^. We studied the neural dynamics in MCC and LFPC, to uncover the mechanisms contributing to decisions to check. We found that MCC has a particular role in encoding decisions to check for information. MCC activity reflects information gathered and used for this decision, and reveal a functional link between the neural substrates of checking and feedback processing. Finally, decision to check is contingent on particular neural dynamics within MCC and LPFC, and contrasts with a typical decision in a cue-based task, suggesting that in decision-making the dynamics within the prefrontal cortex are adjusted to the source of decision.

## Results

### Behaviour

**Figure 1:**
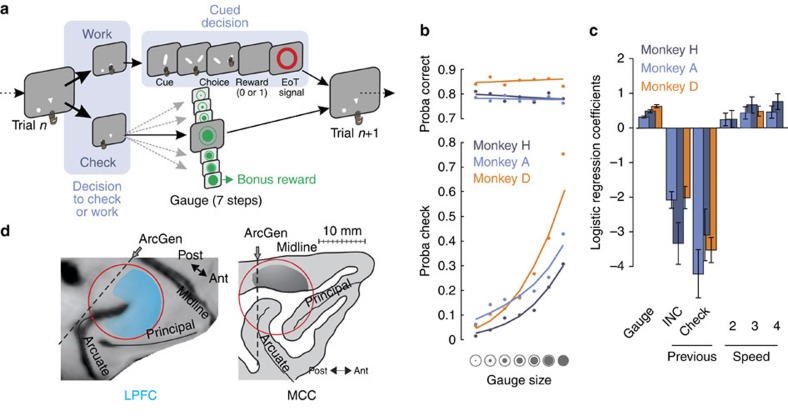
Experimental protocol and behaviour. (**a**) In each trial, monkeys are allowed to freely decide either to Work in a main task (where they are asked to perform a Cued decision) or to Check the gauge state that increases with the number of correct Cued decisions. The speed of increase is based on the total number of correct trials required (four possible speed; see Methods). A check leads to the onset of the current gauge stimulus (green disk with circle). A bonus reward is obtained when checking occurs, while the gauge is full. The gauge is reset after delivery of the bonus reward. (**b**) Monkeys increase check frequency with increasing gauge size (bottom), while keeping stable performance during Cued decisions in the main task (top). Dots are actual data (average over sessions), whereas lines are logistic fits. (**c**) Logistic regressions for each monkey testing the contribution of gauge size, performance in the previous trial (Previous; Incorrect and Check compared with correct) and speed of gauge increase (Speed; compared with speed 1) to the probability of checking. The estimated coefficients notably show a reduced probability to check (negative value) after incorrect trials in comparison with after-correct trials during the Cued decision. (**d**) MRI-based reconstructions highlighting the approximate recording locations in LPFC (blue) and MCC (black) from the genu of the arcuate sulcus (ArcGen).

We designed a behavioural task to assess how monkeys checked for information during ongoing task behaviour. The task design is based on a dual-task principle (Fig. 1a). On each trial, monkeys can either select the triangle stimulus to ‘Work' and perform a delayed visual categorization task (Fig. 1 and Methods, referred hereafter to the main task) or select the disk stimulus to ‘Check' and see whether a bonus reward is available. If they choose to work in the main task, they are shown a cue stimulus (a rightward or leftward bar oriented at various angles) and, after a delay, have to categorize its side of orientation by selecting a target on the right or on the left (this Right versus Left decision is called Cued decision). The main task has three levels of difficulty and requires cue-based decision-making. By contrast, choosing the disk stimulus provides information on how soon a bonus reward will be delivered, as indicated by a visual gauge, which gradually fills up. The bonus is delivered only if the monkey checks when the gauge is full. It is noteworthy that if the gauge becomes full it remains full until the monkey checks the bonus option. Good performance in the main task increases the level of the gauge, bringing the bonus closer to delivery. Specifically, the number of correct trials necessary to get the bonus varies pseudo-randomly in successive blocks (from 14, 21, 28 or 36 trials) and the gauge increases in 7 steps (Supplementary Fig. 1). Thus, one way of improving reward income overall consists of performing well in the main task and also in checking once in a while to gather information on bonus availability. It is noteworthy that no explicit information is given regarding a putative need to check or the value of checking, apart from the gauge presented at the monkeys' request.

Monkeys chose to work in the main task on 87.5% of trials, with good and stable performance. In addition, performance varied significantly between the three levels of task difficulty that we imposed (Kruskall–Wallis (KW) test; monkey A: *P*=1.6e−23; monkey D: *P*=4.3e−12; monkey H: *P*=4.1e−20). Difficulty was controlled by adapting the orientation of the cues to obtain three levels of performance for each monkey. Reaction times (RT) in the main task varied with difficulty (multiple regression, log(RT)=Difficulty (three levels)+Previous performance (COR/INC in previous trial)+Block number (in session); analysis of variance p(Difficulty): monkey A: *P*=2e−16, monkey D: *P*=5.5e−05 and monkey H: *P*=0.036).

Monkeys decided to check for the bonus reward on the remaining 12.5% of trials. As expected, all monkeys naturally increased their checking frequency when approaching bonus delivery (KW test, *P*<1e−27; Fig. 1b,c), but checking did not interfere with Cued decisions; performance and RTs in the main task remained constant whatever gauge sizes (KW test, performance: *P*>0.4; RTs: *P*>0.3). When choosing to check or to work, response times also remained constant across gauge sizes (KW test, Work: *P*>0.08 for all monkeys; Check: *P*>0.2 for A and H), except for monkey D who was quicker to check for larger gauge sizes (KW test, *P*=2.5e−6). The decision to check was faster than to work for monkey A and D (KW test, *P*<5e−8) and slower for monkey H (*P*<0.0001). In addition, monkeys exhibited a response time slowing when choosing to Work following an incorrect choice in the main task whatever the difficulty (KW test, *P*<1e−6).

Overall, the checking pattern resembles that observed with secondary reinforcement schedules, but with specific characteristics^21^. It is worth noting again that a bonus was delivered only if the animal checked when the gauge was full. Logistic regressions revealed that checks occurred mostly after correct trials in the main task, indicating that monkeys understood that the gauge increases only after those trials (Fig. 1c). This behaviour appeared quite early during the learning of the task (binomial test; second session for monkey D, *P*=0.0014; third session for monkeys A and H, *P*<0.003).

**Figure 2:**
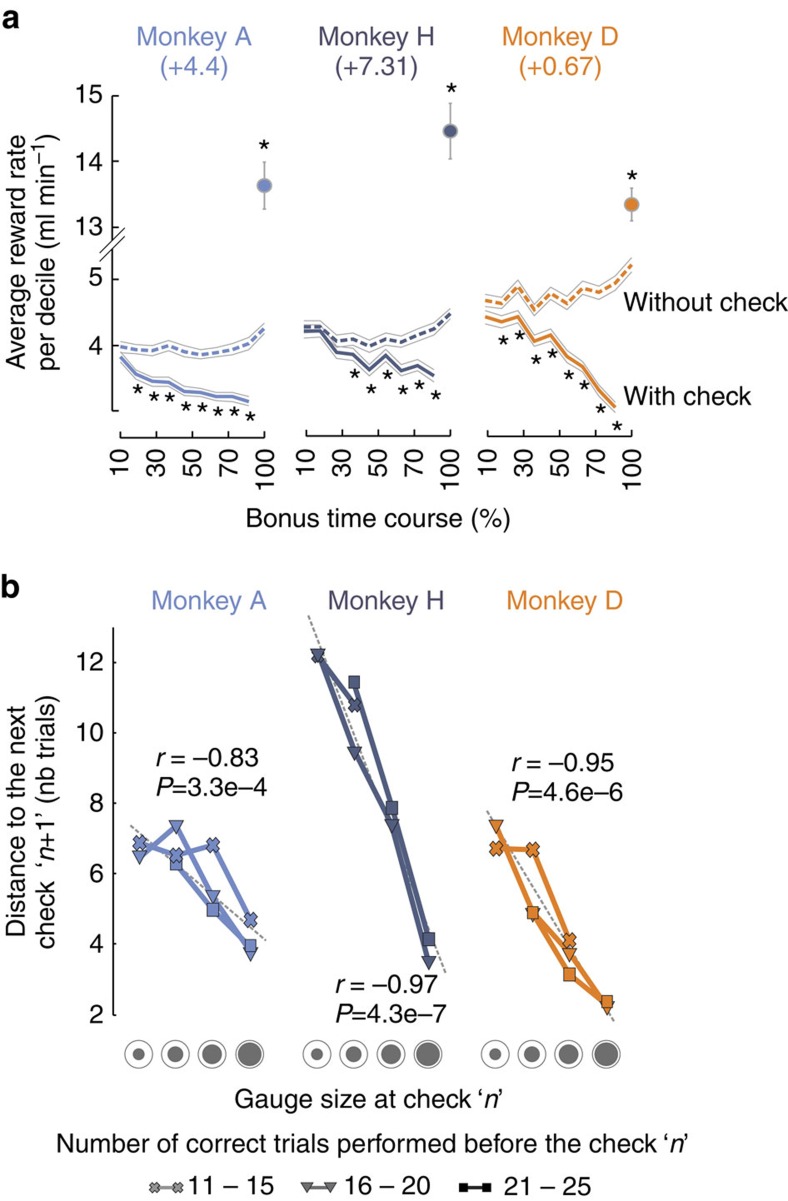
Effects of checking. (**a**) Evolution of average reward rate during a block. Solid lines show for each monkey the average amount of reward per minute obtained during a block (condition: ‘With check'). Each block was divided in ten successive time bins covering several trials. The evolution is expressed in per cent of total time in a block. The single point at 100% corresponds to the last decile that includes the bonus reward. Data can be compared with a hypothetical but ideal case where the animal does not check by excluding the time spent in checking the gauge size (dashed lines, condition ‘Without check'). The number in parenthesis is the difference in cumulated reward rates between the two conditions (‘With check' versus ‘Without check'). It reveals an overall positive gain for all monkeys. (**b**) Effect of observed gauge size on checking. The plots show for each monkey the number of trials (distance) that the animal takes before a check *n*+1 after having seen a particular gauge size (*x* axis) at check *n*. The larger the gauge size seen at check *n*, the smaller the number of trials (distance to check) before the next check *n*+1. The effect is independent of the number of trials performed in the main task in the current block (symbols), that is, independent of time. This suggests that monkeys properly used information gathered from the gauge size to regulate checking. Correlations displayed on graph were made on the three sets of data.

In the short term, checking the gauge size and errors in the main task, both reduce the reward rate. Figure 2a shows that in the course of a block, the reward rate decreases when there is an increase in the frequency of Check decisions (solid lines). This is suboptimal when considering a hypothetical case where monkeys do not check (dashed lines). The loss in reward rate is, however, compensated at the delivery of a reward bonus. Yet, even if unfavourable in terms of reward gain, monkeys often checked for a bonus reward at the very beginning of blocks. Although such behaviour induces a decrease in reward rate, checking the gauge size clearly provides relevant task information. Indeed, the frequency of checks was driven by the information gained from the observed gauge, as seeing a small gauge induced less immediate re-checking than a large one, independently of time or of the number of previous trials performed (Fig. 2b).

Thus, although checking was probably driven by the basic desire to obtain a bonus reward, data suggest that checking also allowed monkeys to seek information and adapt behaviour accordingly, helping to reduce uncertainty^1^. Checking for information also improves accuracy in naturalistic task situations^22^ and our data show that monkeys usually obtained the bonus just after it became available (median=+2 trials). Therefore, a decision to check was driven by motivational and evaluative factors, taking into account the gauge size, performance in the main task and bonus expectation.

### Single-unit and population coding of feedback and decisions

We investigated how MCC and LPFC contribute to decisions to check or work, as well as how they contribute to Cued decisions. We recorded 411 single units in the two structures during performance of the dual task in two monkeys (monkey H and A; 198 in LPFC including dorsal area 8A, area 8B, caudal 9/46d and premotor 6DR (F7), and 213 in MCC in the dorsal bank of the cingulate sulcus; Fig. 1d and Supplementary Fig. 2).

**Figure 3:**
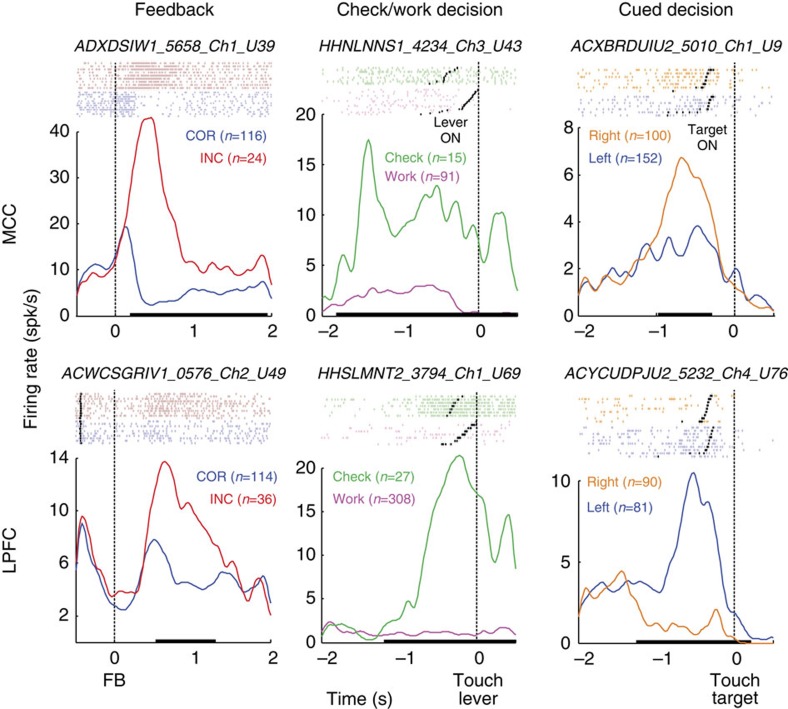
Typical examples of single unit activity. Average firing rates and rasters for six different single unit activities in MCC (top) and LPFC (bottom) discriminating either between negative/positive feedback (left), Check versus Work decisions (middle) and Cued decisions in the main task (right). Black lines on time axis highlight the time period where the difference in firing rate is significant (KW test, *P*<0.01). Only subsets of trials are displayed (*n* represents the total number of trials).

**Figure 4:**
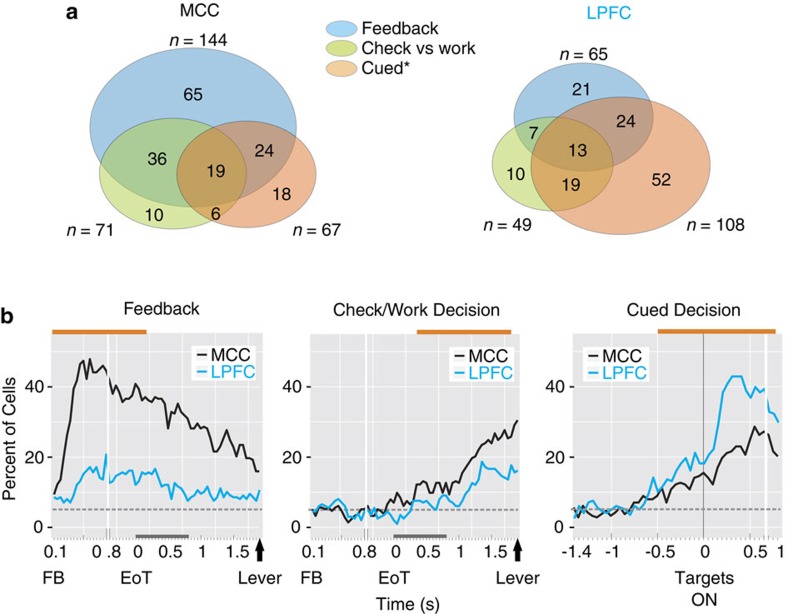
Proportions of neurons encoding feedback and decisions. (**a**) Venn representations of the overall number of neurons discriminating significantly negative/positive feedback, Check versus Work decisions and/or Cued decisions in the main task during the time epochs indicated in orange on top of graphs in **b**. Stars indicate significant differences between MCC and LPFC. (**b**) Time resolved proportion of cells extracted from the sliding *glm* (see ‘Sliding generalized linear models' in Methods) with a significant discrimination of feedback, Check versus Work and Cued decisions. Data presented during the time period between feedback (FB) and lever onset, and aligned on the end of trial signal (EoT, grey bars on the *x* axis represent the duration of the EoT) for Feedback and Check versus Work and on target onset for Cued decisions. Dashed grey lines represent the 5% level.

We first tested whether single units encoded the decision to Check versus Work, the Cued decision in the main task and feedback valence (reward versus no reward in the main task). Examples for single-unit activity are shown in Fig. 3 and Supplementary Fig. 3. Time-resolved generalized linear models (sliding *glm*) were used to extract the proportion of neuronal activity that encoded these different aspects of the task and to determine how these evolved during the task. This initial single-unit analysis revealed a number of differences between areas. Although both were influenced by the different factors, feedback encoding was more prevalent in MCC (MCC=144/213, 67.6%; LPFC=65/198, 32.8%; *χ*^2^=49.6, *P*=1.8e−12), whereas encoding of the Cued decision in the main task was more frequently discriminated in LPFC (MCC=67/213, 31.4%; LPFC=108/198, 54.5%; *χ*^2^=22.37, *P*=2.2e−6) (Fig. 4a and Supplementary Fig. 4). This latter bias was true for almost all lateral subdivisions (Supplementary Fig. 5). Cells discriminating Check versus Work decisions were slightly but not significantly more frequent in MCC than LPFC (MCC=71/213, 33.3%; LPFC=49/198, 24.7%; *χ*^2^=3.65, *P*=0.055).

Inspection of the time course of encoding across the two areas clearly shows the bias in MCC for feedback processing and decisions to Check versus Work (Fig. 4b). By contrast, the bias was reversed in favour of LPFC for the Cued decision in the main task. The selectivity for Check versus Work decisions was not confounded by spatial position or arm movement directions. Among the neurons discriminating Check versus Work in MCC, only 25 also encoded the Cued decisions in the main task (35%) and just 16 (22.5%) did so in a congruent manner (discriminating contra and ipsilateral choices in the same direction). In comparison, 38.8% (19/49) of LPFC units discriminating Check versus Work decisions showed a congruent coding during Cued decisions in the main task (LPFC versus MCC, *χ*^2^=3.7, *P*=0.054). Finally, recordings in a control task, testing for an influence of the spatial position of lever touches, showed very weak, if any, effect on neural activity (see Methods and Supplementary Fig. 6). Thus, in line with the literature, our observations support a weaker role of the MCC compared with LPFC in encoding the spatial component of decisions^11^,^23^,^24^, but see refs 25, 26).

**Figure 5:**
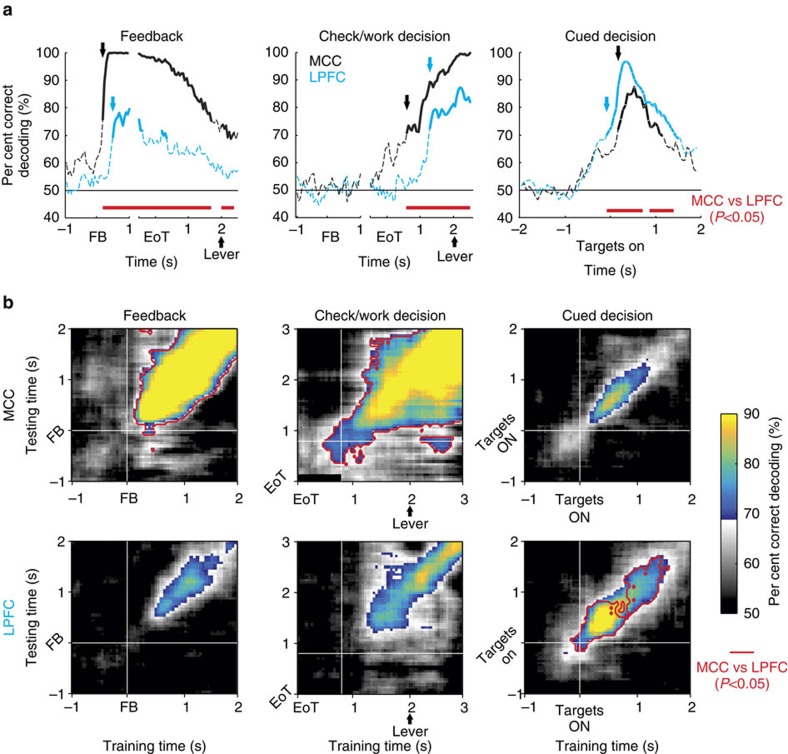
Linear population coding. Linear decoding (**a**) and cross-temporal decoding (**b**) reveal a double dissociation between MCC contributing earlier and more reliably to Feedback processing and Check versus Work decisions, and LPFC contributing earlier and more reliably to Cued decisions during the main task. Arrows in **a** indicate first significant decoding (bold) for each area (colour). Statistical threshold was set at *P*<0.05 using permutation testing. Significant decoding is depicted by bold lines in **a**. Cross-temporal decoding in **b** were thresholded (grey colourmap) depending on the smallest significant decoding rate at *P*<0.05. Statistical comparisons of MCC and LPFC population coding were tested using KW tests. Significant differences (at *P*<0.05, Bonferroni corrected) are displayed in red on the time axis in **a** and within a red contour in **b**. The red line is drawn at the level of the structure with better decoding performance.

We quantified the differences between the two areas at the level of neural populations for feedback, decisions to Check versus Work and Cued decisions in the main task using linear decoding (Fig. 5). The latency at which a decoder could discern task-related factors (Fig. 5a) revealed that MCC leads LPFC in feedback processing, confirming and strengthening previous observations^24^,^27^. Decoding feedback was also much more efficient with MCC activity. However, most importantly, decisions to Check versus Work were decoded from MCC activity before and more efficiently than from LPFC activity. The MCC population activity was predictive of whether the monkey would check well before options were available (before levers' onset). An alternative nonlinear support vector machine decoding method provides similar observations, confirming previous investigations (Supplementary Fig. 7 and ref. 28).

Linear decoding showed that feedback processing dominates in MCC, and that it overlaps in time with the encoding of Check versus Work decisions. The evolution of coding is especially evident when cross-temporal decoding is applied (Fig. 5b), providing a measure of the dynamic of information coding in the population (that is, whether the encoding of a particular dimension is maintained over time). Likewise, it highlights the functional dissociation between MCC and LPFC populations. The restricted diagonal band of significant decoding performance reveals the dynamical nature of neural population coding in both structures and for all events considered. Thus, although linear decoding shows stable discrimination of events over at least a second, the discrimination is computed dynamically. Single units in both MCC and LPFC contribute sequentially to encode the different events, suggesting that integration of information as well as interactions between MCC and other areas should rely on such dynamics.

Importantly, in contrast to feedback and checking for the bonus reward, LPFC led over MCC for the Cued decisions in the main task. The change in lead appeared in terms of strength and also in terms of latencies of decoding (Fig. 5a,b). Thus, relationships between the two regions depend on the cognitive context and this contributes to the neural specificity of decisions to check.

### Specificity of the neural bases of decision to Check

**Figure 6:**
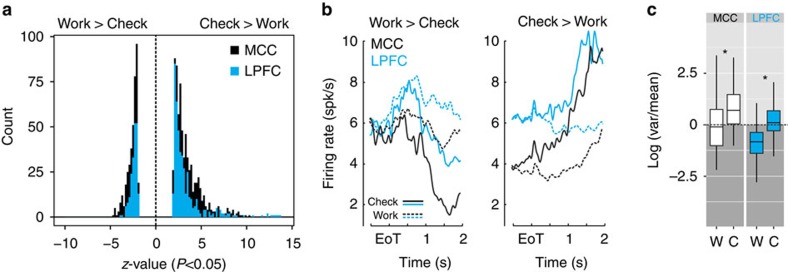
Bias for Check and dynamic of firing rate. (**a**) Both MCC and LPFC show more cases with higher activity in Check compared with Work at decision time. The population histograms show the individual cell *Z*-values from the sliding *glm* for all time bins for all cells (see Fig. 4). (**b**) Average population activity shows for all cases greater dynamical changes for decision to Check (plain lines) than for decisions to Work (dashed). PSTH are displayed for subpopulations of cells that had higher activity in Work than in Check (left), and that had higher activity in Check than in work (right). Data for each decision (Check: lines; Work: dashed lines) and each structure (MCC: black, LPFC: blue). (**c**) Normalized variance of activity across bins is higher for Check than Work in both structures. Stars indicate significance at *P*<10^−3^, analysis of variance on log values (Check versus Work decision × Check versus Work preference).

**Figure 7:**
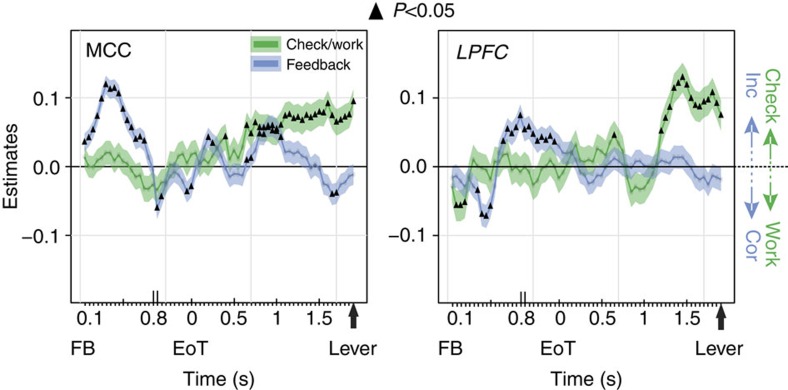
Feedback and Check versus Work specificities at the cell group level. Estimates (β-coefficients) obtained from the MCC and LPFC population, and time-resolved *glm* for Check versus Work (green) and Feedback (blue) variables (see ‘Group analyses using *glmm*' in Methods). Significant effects are indicated by a black triangle (*P*<0.05). Positive values depict a population activity bias towards Check decisions (green line) or negative feedback (blue line), whereas the opposite represent a bias towards Work decisions or Positive feedback.

Deciding to check for information might be qualitatively different from other decisions. Checking appears to be driven by the need to reduce uncertainty or by the rewarding nature of novel information^29^,^30^,^31^. Thus, could checking be the result of a separate type of decision and of specific computations in prefrontal areas? We found that the decision to Check, in contrast to the decision to Work, engaged particular dynamics of MCC and LPFC. First, most of the contrast between Check and Work comes from greater neural activity for Check in both MCC and LPFC (bias towards positive *z*-values from the sliding *glm*; Fig. 6). More units increased activity for Check compared with Work (MCC, 69%, *χ*^2^=11.4, *P*=0.0007; LPFC, 71%, *χ*^2^=9.3, *P*=0.002). In addition, comparatively higher firing rates for Work (negative *z*-values) corresponded in reality to a reduction of activity during Check. This resulted in an increased bin-to-bin variance of firing during decision to Check compared with Work (Fig. 6b,c). Therefore, changes in neural activity did not occur for the default decision to Work. Using a mixed model (*generalized linear mixed-effect model* (*glmm*), see Methods) as a group analysis to quantify the influence of various task variables in the population of activity, we confirmed this bias towards decision to Check (represented by positive estimates; Fig. 7). This was true for both areas, although the time course differed. As previously observed (for example, see refs 32, 33), MCC units processed both positive and negative feedback, with a slight bias towards greater activity for negative feedback (early positive estimates; Fig. 7).

The statistical estimates from the groups and individual neuron statistics (Fig. 4) suggest that multiple neuronal populations contributed to different events. In particular, the strong representation of feedback in MCC activity involved neurons that also encoded the Check versus Work decision (77.5%). In the MCC, 56.3% of units that encoded decisions to Check versus Work also coded for negative feedback, whereas 21.1% coded for positive feedback. We further investigated relationships between parameters encoded by neuronal activity and found that only Feedback and decisions to Check versus Work were correlated (correlation of absolute maximal *Z*-values over four successive bins obtained from the sliding *glm* and used in Fig. 4a, Feedback and Check versus Work: *r*=0.24, *P*<0.0005). This relationship was not significant in LPFC. It is noteworthy that all effects were confirmed using an alternative method based on hierarchical clustering (see Methods and Supplementary Fig. 8).

The data hence highlight in the MCC a clear functional relationship between feedback and decision to Check. In contrast, the LPFC was characterized by the coding of Cued decisions in the main task with a bias towards encoding the spatial elements of decisions.

### Encoding gauge value and distance to check

**Figure 8:**
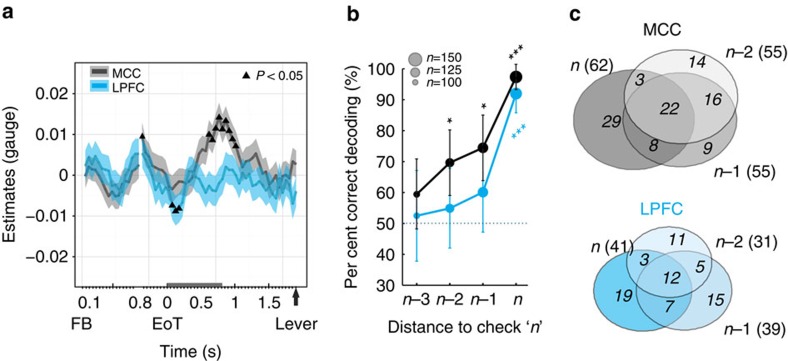
Encoding of Gauge size and Distance to check. (**a**) Estimates (± CI) for the variable Gauge extracted from the time-resolved group *glmm* (as in Fig. 7). Significant effect of gauge size is indicated using triangles on data for each structure (*P*<0.05). The grey bar on the *x* axis represents the duration of the EoT signal. (**b**) Average per cent of correct decoding between trials at short distances to Check and trials at long distances using inter-trial firing rates (mean±s.d.). The circle size represents the number of neurons used by the decoder (see Methods). Dotted lines represent the chance level calculated from permutation testing and statistical differences are displayed using asterisks for each structure separately (*P<0.05 and ***P<0.001). (**c**) Venn representations of the number of neurons contributing to the decoding (including neurons with non-zeros coefficients in >10% of trial permutations; see ‘Population analyses' in Methods) of trials *n*, *n*−1 and *n*−2 against those at longer distance to check (as in **b**). It is noteworthy that in MCC the subpopulation of neurons contributing to decoding both *n*−1 and *n*−2 was larger than the one contributing also to decoding trial *n* (*χ*^2^-tests, *P*<0.05).

The monkeys' behaviour suggests that the information used for deciding to check includes time, information gathered from previous checks and performance in the main task. Integration of those variables could take place after key task events (for example, following performance feedback), or be spread and maintained across trials, thanks to the dynamical nature of prefrontal coding^14^ or to sustained states of the executive system^34^. We thus sought traces of gauge information in neural activity between trials and within the main task, testing the hypothesis that even Cued decisions in the main task would incorporate current estimates of the gauge state. To do this, the independent variable *Gauge* was included in statistical models devoted to inter-trial and to the main task periods. Although information about gauge seemed weakly represented during the main task (12% of neurons in MCC and LPFC at target touch in the main task), we did not find clear evidence of such coding in the group *glm* at this time period (only two non-consecutive bins showed an effect at *P*<0.05, none at *P*<0.01). However, the gauge size appeared to be significantly and transiently encoded positively between trials, especially in the MCC group of cells, and in both monkeys (Fig. 8a and Supplementary Fig. 4c). Importantly, gauge encoding occurred at about the time of Check versus Work discrimination (see Fig. 7). This suggests that gauge size contributes to the elaboration of decisions to check in MCC.

We then hypothesized that check decisions might be made dynamically based on evidence accumulated from successive trials and previous checks. If this were true, population activity would show progressive evidence towards a decision to check across trials preceding the actual choice to check. Decoding population activity showed that MCC and LPFC activity during the inter-trial period, when monkeys were just about to check, robustly differed from other inter-trial periods (Fig. 8b), confirming our previous observations (Figs 4 and 5). However, most importantly, MCC population activity during the two trials preceding a check was significantly different and one could predict the monkeys' decision to Check even a few trials before. The early check-predictive activity (at *n−*1 and *n−*2) was significant in MCC and only marginal, although with a similar trend, in LPFC. Importantly, the regression coefficients for each neuron significantly involved in the different decoding outcomes were well correlated (correlating coefficients from *n* versus *n−*1, *n−*1 versus *n−*2 and *n* versus *n−*2: all *R*^2^>0.4, *P*<0.0005), suggesting a stable and consistent population code over trials preceding a decision to Check. Yet, such stable encoding was driven by a subpopulation of MCC neurons partially different from an additional one contributing to the actual choice of Checking (at trial *n*) (Fig. 8c), suggesting a neural dynamic specific to the actual choice of checking. The later point (that additional activity was specific to the actual implementation of the decision to check) was confirmed by alternative decoding, which clearly differentiated trials *n* from trials *n−*1 or *n−*2 (MCC: accuracy>84.3%, *P*<0.0005; LPFC: accuracy>89.1%, *P*<0.0005).

Although little activity variation was found during decisions to Work, neural population activity differed within the two trials preceding a check. Such difference might reflect increased uncertainty about whether to Check or Work, while approaching the actual choice. Response times to select the Work option do not support an effect of uncertainty, as they remained stable across trials before a Check (Supplementary Fig. 9). However, the task design allows to plan decisions before the onset of levers, making these response times unreliable. We thus investigated free oculomotor activity during inter-trial periods, before the levers' onset. Fixations on levers' positions varied from trial to trial and provided relevant information. We retained three major measures: scanning, that is, whether or not the animal fixated alternatively between the two lever positions, the latency between the last fixation on a lever position and the lever touch, and the number of fixations on both levers when scanning. Scanning the two lever positions might reflect hesitation or consideration to the two options. A late final fixation (that is, closer to touch) can be interpreted as longer deliberation and thus higher uncertainty or consideration of the options, whereas making the last fixation early might reflect a low level of uncertainty. We tested with multiple regressions whether these measures varied with the gauge level, distance to check and with the decision to Check versus Work.

First, monkeys scanned both levers in 18% of trials and were less likely to do so, while approaching bonus delivery (logistic regression, fixed effect gauge size: monkey A: Estimate=−0.29, *P*=0.0007; monkey H: Estimate=−0.30, *P*=0.0068). The probability of scanning slightly increased while approaching the next decision to check for the bonus reward, but only in monkey A (*P*<0.001; *P*=0.44 for monkey H). Neither latencies of the last fixation nor the number of fixations changed significantly with gauge size. However, all three variables were changed before choosing to Check compared with Work. When deciding to check for the bonus, monkeys were more likely to scan both levers (fixed effect of Check versus Work, *P*<10^−8^ for both monkeys), performed fewer fixations (Poisson *glm*, fixed effect of Check versus Work: monkey A: Estimate=−0.12, *P*=0.0016; monkey H: Estimate=−0.13, *P*=0.0032) and ended fixation much closer to the lever touch (fixed effect on latency: *P*<10^−8^ for both). Overall, these data suggest that monkeys were more likely to consider the two options (Check or Work) at the beginning of a block than when close to the full gauge, but at the same time they were more likely to scan just before each decision to Check. Check versus Work decisions were thus influenced in two ways, across a block and before each decision to check or not, the time for decision being longer just before a check.

We further tested whether the odds of scanning levers by the eye could explain part of the variance in the group neural activity in MCC and LPFC. The mixed model revealed that indeed Scan, Gauge size and Check versus Work decision effects were contributing to variation of the group activity but at different time before the actual lever touch (Supplementary Fig. 10). In particular, Scan and Gauge influenced neural activity before decision to Check or Work did. No interactions between Scan and Check versus Work factors were found at any time bin.

Finally, as difficulty was a parameter within the main task (effect of conditions on performance and RTs), we assessed its contribution to neural activity variations. The sliding *glm* revealed effects in a number of cells as expected by chance (Supplementary Fig. 11a). The group *glmm* revealed an effect of difficulty in monkey A only between cue and target onset. We finally tested whether the strength of Check versus Work effect (absolute *z*-value in the sliding *glm*) would correlate across units with the effect of condition difficulty in the main task (absolute *Z-*value for Difficulty). We found no correlation in MCC (*r*=0.005, *P*=0.95) or in LPFC (*r*=0.04, *P*=0.56) (Supplementary Fig. 11b).

## Discussion

We report three major results. First, MCC and LPFC neural dynamics related to decisions to check the environment is specific, suggesting network reconfigurations depending on decision types (Check versus Cued). We further demonstrate that the encoding of decisions to check has a link with feedback encoding. Finally, we show that the activity related to decisions to check steadily builds across trials in MCC.

Checking on the current state of the environment might be driven by the need to reduce uncertainty or by the rewarding nature of novel information^29^,^30^,^31^. Here, although probably initially driven by the desire to get a bonus reward, checking quickly became an efficient strategy to obtain information on the timing of bonus availability. One drawback of our task design is that the delivery of the bonus reward requires using the checking option. One can argue that checking at any time is driven not by the will to gather information on the gauge but rather by the hope of gathering the actual juice reward. Future experiments will require an explicit separation between bonus delivery and checking, but although the use of gauge information does not in itself imply checking was driven by information seeking, altogether behavioural data suggest this was the case. In particular, checking occurred only after correct trials (the only events causing gauge increase), checking frequency was regulated based on the observed gauge size and the bonus was obtained close to its availability. This suggests that although checking is costly, animals used it to track the gauge speed, reducing uncertainty and adjusting to get the bonus as soon as possible. Information seeking or foraging might be qualitatively different from other decisions and rely on particular networks and neural activity. This is indeed supported by our findings showing particular neural coding and dynamics in MCC and LPFC, with MCC leading both in strength and time during checking. Overall, no equivalent changes in neural activity occurred for selecting the main task; the most prominent neural changes were when animals decided to check. One possibility is that check-related prefrontal activity reflects not only the orientation towards information seeking but also an active mechanism to disengage and shift from the routine conduct, that is, from choosing the main task.

The particular involvement of MCC in decisions to Check versus Work appeared through every analytical approach. Strikingly, increased MCC activity for checking was mostly produced by neurons also involved in coding feedback and, in particular, negative feedback, and was only weakly involved in coding spatial information. This result suggests a functional commonality between feedback and decisions to check as far as the MCC is concerned. The co-location in MCC of activity modulated by exploration or volatility and of feedback-related activity has been reported previously^33^,^35^. Here, however, statistics and classification of neural activity unveils a direct structural and functional similarity. Such combination reflects the link between monitoring relevant outcomes or feedback of actions and the computations leading to exploratory decisions^33^,^36^. Feedback indeed is a major source of evidence contributing to ruptures in behaviour, for shifting responses or strategies. This reinforces again an interpretation of check-related activity as a correlate of a mechanism to depart from a default option.

Although the type of behaviour we studied here does not directly relate to abnormal checking observed in obsessive compulsive disorders (OCDs), one must note that MCC might have a direct or indirect role in abnormal and compulsive checking^19^,^37^,^38^. Some of the major symptoms of OCD are classified as aggressive/somatic obsessions with checking compulsions, which can notably be interpreted as the emergence of misperceptions that ‘something is wrong' in a specific situation^39^. These symptoms might correspond to overactive performance monitoring^37^ and/or to an abnormal production of default and automated behaviours^40^. Enhanced error-related activity and reduced feedback-related negativity, whose main source might be in the MCC^7^, have often been observed in OCD patients, although not systematically and with unclear behavioural counterparts in traditional cognitive tasks^37^. Our data clearly reveal a link between feedback processing and checking, and suggests that by using a suitable task incorporating both performance monitoring and checking, and by focussing on specific mechanisms (for example, disengagement from default option) occurring at the times of check decisions, clearer links between monitoring and checking might emerge in pathological cases.

One must emphasize two characteristics of our design. First, contrary to previous protocols^12^,^33^,^41^, we studied purely voluntary decisions to check, because no direct objective feedback or observable cue was provided to inform on the potential value of exploration or on the need to explore. Second, the time scale at which incentives to check are built crosses over several trials and encompasses two intermingled tasks, thus making the integration time more naturalistic, even if prone to interference, compared with previous tests^18^.

Medial prefrontal areas and MCC activations might index multiple signals leading to a decision to explore new alternatives, including the value of the ‘explore' option and the difficulty in deciding for a non-default course of action^10^,^12^,^13^. In our task the average value of the default Work option is considered stable, because performance in the main task was constant across blocks. However, the gauge level can be taken as a proxy for the value of the Check option (the fuller the gauge, the closer the bonus availability). Alternatively, difficulty, uncertainty in decision and increased deliberation time might be reflected in the oculomotor patterns of monkeys just before Check versus Work decisions. In fact, we showed that scanning the option levers was negatively correlated with gauge increase, but was also more probable just before a check.

At the neural level, gauge-related information did appear clearly at the time of decisions to check or not. Population data suggest that a momentary re-instantiation of gauge information occurs just before the build-up of activity related to the decision to Check or Work. This phenomenon might contribute to adapt checking behaviour. Separate statistics for Check and Work decisions showed that gauge information was present in both cases. Importantly, scanning targets by the eyes also contributed to the modulation of neural activity before the decision, separately from the gauge levels and from the actual decision to check. The timing of these events might suggest that scanning alternatively lever positions could contribute to the re-instantiation of gauge information, which would then inform the decision to check or not. Thus, MCC activity reflects indeed multiple signals contributing to the decision to explore, both across trials and all along the elaboration of that decision^13^. The neural dynamic might thus reflect a series of mechanisms engaged for the decision to check. However, it is still unclear whether these mechanisms and the longer time taken for a decision to check include the actual process of active oculomotor exploration, an increased uncertainty and/or a phenomenon related to decision difficulty. It is also noteworthy that recently some specific MCC activity modulations have been interpreted as reflecting task conflict^42^. We attempted to relate the neural contribution to check to the effects of uncertainty or difficulty in the main task without success. The contribution of oculomotor scanning in the decision process might reflect the specific recruitment of the rostral face/eye representation of the MCC to control behaviour and to gather information for adaptive decisions^7^. Future experiments will need to explore the specific role of MCC body representations in decision making.

Altogether, the data suggest that MCC activity reflects the progressive integration of cues to build a decision to check and, through different but overlapping neural populations, the actual engagement in checking.

The neural coding specific to exploratory decision reveals the relative contributions of MCC and LPFC, and MCC is clearly leading the process. Importantly, although both areas contribute to different events of the task, the temporal lead of activity variations and reliability of information coding between the two regions dynamically change depending on the current type of decision. This is remarkable, as most previous studies, including ours, have focussed on a hypothetical sequential process from MCC to LPFC^11^,^27^,^43^,^44^. It emphasizes a flexibility of the relative recruitment of frontal areas possibly depending on the source of evidence contributing to decisions. Interactions between areas might occur through spike-field and cross-frequency coordination^45^. Whereas LPFC might receive input from MCC to implement exploratory decisions, input from other areas (for example, parietal areas) might lead in case of externally cued decisions^46^. Our work suggests that MCC's influence over LPFC takes place only when internal drives and reinforcement-based information are the major contributors to behavioural adaptation. It further suggests that specific large-scale functional network configurations, possibly involving the orbitofrontal cortex and amygdala (key structures for decision-making and adaptation, and linked to OCD^38^), might contribute to the regulation of exploratory checking behaviours. The notion that network reconfigurations support different decision types indicates that the core network described as common to various cognitive tasks must be seen as a flexible, and not a stable, entity that is engaged qualitatively differently in various cognitive activities.

## Methods

### Subjects and materials

This project was conducted with three male rhesus monkeys (*Macaca mulatta*), three for behavioural analyses and two for neural recordings. Monkey A was 16 years old and monkey H was 8 years old at the time of experimental sessions and were included in the recording part of the experiment. Monkey D, 8 years old at the time of experimental sessions, performed only the behavioural part.

All procedures followed the European Community Council Directive (2010) (Ministère de l'Agriculture et de la Forêt, Commission nationale de l'expérimentation animale) and were approved by the local ethical committee « Comité d'Éthique Lyonnais pour les Neurosciences Expérimentales », CELYNE, C2EA #42. Monkeys were habituated to sit in a primate chair (Crist Instrument Co., MD, USA) in front of a tangent touch screen monitor (Microtouch System, Methuen, USA). An open window in front of the chair allowed them to use their preferred hand to interact with the screen (monkey A and D, left-handed; monkey H, right-handed). The position and accuracy of each touch was recorded with the computer in charge of visual stimuli presentation (CORTEX software, NIMH Laboratory of Neuropsychology, Bethesda, MD). Eye movements were monitored and recorded using an Iscan infrared system (Iscan Inc., MA, USA; sampling rate=2.8 kHz). Electrophysiological data were recorded using an Alpha-Omega multichannel system (AlphaOmega Engineering, Israel). Juice reward was provided by a computer-controlled system (Crist Instrument Co.).

Monkeys were implanted with a head-restraining device in a first surgery. Next, in a second surgery, a craniotomy was performed to expose an aperture over the prefrontal cortex and a recording chamber was implanted (Gray Matter Research, MT, USA) (see below Recordings). Analgesics and antibiotics were administered throughout surgical periods. The chamber was kept sterile with regular cleaning and sealed with sterile caps.

### Behavioural tasks

Monkeys were trained to perform a dual task motivated by juice reward. At the beginning of each trial, monkeys had the opportunity to either work or check their ongoing progress (Check versus Work choice), by touching and holding for 700 ms one of the two ‘lever' targets (triangle to Work and circle to Check; Fig. 1). Levers were always displayed at the same positions (laterally aligned on the centre of the screen for Work and contralateral to the arm used for Check). This was to ensure that when a decision to check was made it was not just for a question of ease of touching a closer position on the screen. A dot appeared at the centre of the screen during lever holding, for both options, but gaze fixation was never required. Stimuli were all presented on a grey background screen.

The Work option consisted in a visual categorization task (referred as the main task) based on the orientation of a stimulus. Monkeys were presented a central smoothed oriented bar (600 ms for monkey A and D, and 350 ms for monkey H) followed by a 400- or 650-ms delay (for monkey A/D and H, respectively). The two targets were then presented, consisting of two oriented bars (±45° from vertical plane) presented on either side of the screen and, to get a reward, the animals had to touch the target oriented in the same direction than the previously presented stimulus (Cued decision). For all monkeys there was 1,000 ms between the onset of the stimulus and the target onset. The relative positions of targets were randomized from trial to trial for monkey H and D. Monkeys responded by touching one of the two targets and after a random delay interval of 200–600 ms (step of 200 ms), a central dot was presented for 500 ms. Cued decisions in the main task were either rewarded (50% water/apple juice, 0.5 ml) if correct or penalized with a timeout (2,000 ms) when incorrect. After feedback onset (1500 to 2,000 ms), an End of Trial (EoT) signal (red circle on for 800 ms) signalled that a new Check versus Work choice would be presented after a fixed delay (700 or 1,000 ms for monkey A and H/D, respectively).

We varied task difficulty by using one of three possible absolute orientations (relative to the vertical axis) of the cue, which was pseudo-randomly selected for each Cued decisions (in total, three oriented rightwards and three leftwards). Orientations were not fixed between sessions, but varied depending on monkeys' performance in previous sessions (using the five to ten last sessions). At the start of each session, we computed a psychometric curve from the monkey's previous performance using a binomial (logistic regression) generalized linear model. Next, stimuli orientations were selected to elicit 70, 80 or 90% of correct responses on average. These three orientations thus corresponded to three levels of difficulty. This allowed us to maintain uncertainty on Cued decisions during the main task, even if monkeys' performance changed between sessions.

Instead of selecting the Work option, monkeys could select the Check option. Here, when monkeys selected and maintained the Check lever, a gauge was displayed for 800 ms, indicating how far they were from a bonus reward. If the Check option was chosen while the gauge was full, a bonus reward (3.5 ml; 7 times a reward in the main task) was delivered 400 ms after gauge appearance, otherwise (in case of partial gauge) the trial ended without reward or penalty. After a delay of 1,800 ms (for monkey A and 1,500 ms for monkeys H and D), the next trial was proposed.

The gauge was filled according to the number of correct Cued decisions performed in the main task from the beginning of a block. Incorrect choices or execution errors during the main task did not affect the gauge evolution. The number of correct choices required to complete a block (and to obtain a bonus) was randomly selected and could either be 21 or 31 for a first set of behavioural sessions or 14, 21, 29 or 36 for a second set of behavioural sessions (Supplementary Fig. 1). Monkey D was trained only during the first set of sessions. For monkeys A and H, behavioural analyses and electrophysiological recordings were performed only with the second set of sessions. Monkeys showed adapted behaviour in both cases (data not shown), allowing us to consider equally all sessions. Once monkeys reached the required number of correct trials in a block, the bonus reward stayed available until the next check (that is, Bonus rewards could not be lost by performing too many trials in the main task). After a bonus reward was delivered, the gauge size was reset and a new pseudorandom number of required correct choices in the main task was picked for the following block.

The gauge was represented by a large green circle, containing a plain, filled green disk. The circle indicated the maximum to be reached (see symbols in Fig. 1a,b and Supplementary Fig. 1). The full gauge size associated with the bonus reward was represented by a full green disk with a diameter identical to the outside circle. The diameter of the green disk could take seven different values, increasing linearly depending on (1) the number of correct Cued decisions performed in the main task and (2) the total number of trials required to earn a bonus (see Supplementary Fig. 1).

In addition, on 15% of trials, monkey A was forced to touch a square lever, which was presented alone and pseudorandomly at one of the spatial positions of the Check and Work levers. The monkey earned no reward during this Control task. This allowed us to control whether Check versus Work choice neuronal activity was modulated by the spatial property of levers independently of confounding factors related to decision difficulty and/or reward expectations.

### Recordings

Recording chambers were centred at antero-posterior coordinates of +34.4 and +33.6 relative to ear bars (for monkey A and H, respectively). Electrophysiological activity was recorded using epoxy-coated tungsten electrodes (1–2 MΩ at 1 kHz; FHC Inc., USA) independently lowered using microdrive guidance (AlphaOmega Engineering). Neuronal activity was sampled at 22 kHz resolution. Recordings were referenced on guide tubes containing the electrodes and in contact with the dura. Two to six electrodes were used simultaneously to record neuronal activity in MCC and LPFC. The label MCC is used to emphasize the homology of the recorded region with the aMCC in humans, where feedback-related activation have been observed—see Procyk *et al.*^7^ for details.

Recording sites (see Supplementary Fig. 2) were reached through a 1-mm spaced recording grid and covered in MCC, an area extending over ∼10 mm (anterior to posterior), and at depths superior to 4 mm from cortical surface. Recording sites in LPFC were located between principal and arcuate sulcus (areas 6DR, 8B, 8A and 9/46) and at depths inferior to 2 mm from cortical surface. Regions were targeted pre-operatively using Brainsight neuronavigation (Rogue Research Inc., Canada) using each monkey's anatomical MRI (T1, 1.5 T and voxel 0.6 mm iso). Recording chambers were implanted contralateral to the arm used with online targeting using neuronavigation. Reconstructions of cortical surface, of MRI sections perpendicular to recording grids and of microelectrode tracks were performed using the same reference frames. Maps and recording locations in the two monkeys were combined by aligning grids on the level of the genu of the arcuate sulcus, taken from coronal sections on the MRI and then relocated using views perpendicular to the recording chamber axis. Locations were confirmed with those MRI reconstructions and stereotaxic measurements by keeping track of electrophysiological activity during lowering of electrodes.

### Behavioural analyses

Only sessions with more than four earned bonus rewards (four blocks) were included. We used 48, 44 and 26 sessions to investigate monkeys' behaviour during the dual task (in monkey A, H and D, respectively). Break trials and trials of the control task were not used for behavioural analyses.

RTs (time between the appearance of the two targets in the main task and the release of the lever) and movement times (time of arm movements from lever to target) were computed on each Cued trials in the main task. For the Check versus Work decisions, response times (time between the lever onset and the lever touch) were also recorded and analysed.

A logistic regression was used to establish the impact of events on the odds of choosing the Check option in contrast to the Work option. *glm* were fitted using R^47^ and the packages MASS^48^, as well as ggplot2 for graphics^49^.

The main *glm* used to explain the odds of checking included the following variables: Gauge (representing the gauge size, from 1 to 7)+Previous performance (Incorrect/Correct)+Speed (representing the various number of correct trials defining the gauge size increase, from 1 to 4)+Block number (order of the block in a given session, from 1 to 10). The significance and estimates were extracted: slope for Gauge and difference from Correct estimate for Previous trial, and difference from speed 1 estimate for Speed. Block number provided no significant effects and is thus not presented in Fig. 1c.

Similarly, a *glm* was used to explain possible variations of RTs in the Cued decision during the main task. The *glm* included the following variables: Difficulty (representing the orientation of the cue, from 1 to 3)+Previous performance+Block number.

### Oculomotor analyses

Oculomotor signals were first subsampled at 186 Hz for ease of data processing. A cleaning procedure using robust spline smoothing with generalized cross-validation to minimize over- or under-smoothing automatically was then applied to remove blinks and interpolate short periods of poor signal/noise ratio (<20% of the trial length). Trials with longer noisy periods were removed from the following analyses. It is noteworthy that sessions considered here are parts of the recording set as we wanted to use oculomotor measures as a factor to explain neuronal activity modulations.

Oculomotor signals were extracted from 1,000 ms before the EoT and until monkeys' touch on one of the lever (Check or Work) in the following trial. We then defined non-overlapping windows around the spatial position of each lever (2.5 times the stimulus lever sizes) and detected when monkeys' gaze were inside either windows of interest. Only fixations of one of the levers for >150 ms where considered. We also counted the number of fixations on each levers during the time period of interest. In that case, two successive fixations needed to be separated by >150 ms to be considered.

Three measures were then considered. The scanning behaviour (named Scan) corresponds to the fact that the animal fixated either only one lever position (0) or both lever positions (1) during the inter-trial period. Furthermore, we extracted the time of the last lever fixation after the EoT and up to the touch. It is noteworthy that the latencies were realigned to the touch latencies instead of the EoT onset. This measure represents an alternative to using the Response time for check or work decisions by providing information about the relative time at which monkeys last considered which decision to make. Finally, we considered the total number of fixations performed in cases were monkeys scanned both levers (that is, when Scan=1).

We analysed, using generalized linear models, the fixed effects of Gauge size, Distance to check, Previous performance (Incorrect/Correct) and Check versus Work decisions on the three oculomotor measures (Scan, Latency and Number of fixations) using binomial, normal and Poisson link functions, respectively.

### Spike sorting and visualization

Neuronal activity was visually inspected using online spike sorting (MSD, AlphaOmega) and then analysed offline using spike sorting (UltraMegaSort2000, Matlab toolbox, Kleinfeld Lab, University of California, San Diego, USA; see also ref. 50). We referred to metrics qualities to verify the completeness and purity of unit activity and thus to infer the presence of single neurons. Each single unit activity was selected, recorded and included in analyses on the basis of the quality of isolation only. For visualization purpose, we computed the spike density histogram relative to the appropriate conditions for each isolated single unit (convolved using a 20-ms Gaussian kernel) and averaging activity using a sliding window of 20 ms each 20 ms step (Matlab, The MathWorks Inc., home-made scripts).

### Sliding generalized linear models

To investigate whether each single unit activity encoded the different key variables of the task, we analysed variations of spike counts measured in each trial using a *glm* approach on the R platform, the libraries MASS and ggplot2 for graphics (see above) (Fig. 4).

Spike counts were measured on successive bins of 200 ms moved smoothly by 50 ms around different key event times in each trial. Because of the statistical properties of count data, the *glm* were applied using a Poisson regression (Poisson error structure). However, evaluation of Poisson *glm* is based on the assumption that the dispersion parameter is equal to 1. The model is inadequate if data are overdispersed (when variance is much larger than the mean). Inappropriate imposition of the Poisson might underestimate the s.e. and overstate the significance of the regression parameters, therefore inducing misleading inference on the regression parameters. One way of checking for overdispersion is to divide residual deviance by the degree of freedom. This should be close to 1 for correct dispersion. In case of overdispersion, we applied Negative binomial regression using the *glm.nb()* function. To validate this choice for each set of data, we statistically compared the two models (Poisson and Negative binomial) fitted for each set (likelihood ratio test, *χ*^2^-test).

Proportions of significant single units were extracted from the sliding *glm* if they significantly discriminated the factor of interest for four consecutive bins (covering a time period of 350 ms) within a pre-defined epoch of 1,500 ms around the event considered (epochs represented in orange horizontal bar on top of graphs in Fig. 4b). Neuronal preference was then defined depending on the sign of the *Z*-value during this period. We assessed statistical significance by computing *χ*^2^-tests when testing for differences in neuronal proportion between structures (at *P*<0.05).

Two *glm* models were used for three different alignments on events. One model was used for activity aligned on both feedback and EoT events, incorporating the variable Feedback (Correct versus Incorrect), Next decision (Check versus Work) and Gauge (covariate 7 steps). For the study of information processed during Cued decision in the main task, we analysed single trial activity aligned on Cue onset or Target touch with one model incorporating the variables Space (Right versus Left choices), Difficulty (1/2/3 angles in the cue-based categorization) and Gauge (covariate 7 steps).

We also checked for multicollinearity with the variation inflation factors (VIF) for each fit (each neuron and each time bin). The VIF provides an index of how much a variable relate to the other variables. It should be 1 for totally orthogonal vectors. A VIF between 2.3 and 5 is supposed to indicate some significant but manageable collinearity, whereas VIF>10 indicates problems and a need to act on variables. We computed VIF for all three variables and found an average VIF of 1.67 s.d.±0.068 (median: 1.04), suggesting collinearity was not an issue in our primary model.

### Group analyses using a *glmm*

The capacity of groups of single unit activity to encode factors were tested using a so-called population *glm*, which correspond to a *glmm*, using a Poisson regression, with single unit considered as random factor. The mixed models used were of the form:


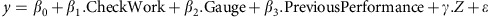


where *γ*.*Z* is the random term, and CheckWork, Gauge and PreviousPerformance are the fixed effects describing the Check versus Work decision (0/1), the gauge size (1–7) and the performance in the previous trial (0/1) with their respective parameters (*β*). To approach the population of single units as a whole, we considered units as a Random factor, hence providing to the model a freedom to take into account single-unit activity individual variance. In the *glmm*, the Single unit identity was used as a random factor, to take into account the potential difference/variance of fixed effects from one cell to another. The analysis takes into account that slopes and intercept differences can vary between single units, as if we were testing effects on a population of different subjects. We add a random effect for ‘units' and this characterizes idiosyncratic variation that is due to individual differences.

A persistent problem with Poisson models in biology is that they often exhibit overdispersion, where the variance of the response variable is greater than the mean. Not accounting for overdispersion can lead to biased parameter estimates; it can bias both the means and s.e. of parameter estimates. One manner in which overdispersion is dealt with involves the use of observation-level random effects (OLRE), which model the extra variation in the response variable using a random effect with a unique level for every data point^51^. The procedure is to create a variable with one level for each single data point, in our case for each spike count measure for each cell, each time bin and each condition. This OLRE variable is used as a random effect. This allows to include in the model the extra variance missing to the Poisson model.

Data observation and model validation revealed overdispersed data overall. To model the excess variation and solve the problem of underestimated mean and s.e., we included an OLRE that showed to improve model performance. We tested the models' fits with and without the OLRE and found that deviance and akaike information criterion (AIC) were always better with OLRE (see Supplementary Fig. 12). Statistical comparisons between models were always significant in favour of the model with OLRE.

The impact of Scan on the group neural activity was evaluated using a glmm with single unit as a random factor and an OLRE to solve overdispersion problems. Fixed effects were Previous performance, Gauge, Scan and Check/Work.

### Hierarchical clustering analyses

The statistical estimates in the group and single-unit activity statistics (sliding *glm* in Fig. 4) suggested that multiple cell populations contributed to different events. To study further the functional typology of single-unit activities, we proceeded to classify units using another approach beyond the one reflected in the Venn representations (Fig. 4).

We applied a hierarchical clustering method to the *Z*-values obtained for each cell and each event (Feedback, Check versus Work decisions and Cued decisions in the main task), to extract objective classes of neurons in both areas. This method complements classic tools used to characterize neuronal preference without the bias of reporting strict categories of neurons. As for Venn representations in Fig. 4a, we used here the maximal *Z*-value extracted from the sliding *glm* during a defined time window (orange bars in Fig. 4b) for each significant neuron and for each variable of interest (Feedback, Check versus Work decisions and Cued decisions in the main task). Values for nonsignificant neurons were set to zeros. It is noteworthy that positives *Z*-values mean a greater activity for Correct choices, Check decisions or Right cued decisions compared with Incorrect choices, Work decisions or Left cued decisions, respectively (negative values therefore represent the opposite pattern). The distance measure used was the Euclidean distance and the Ward's method was used for the aggregation process. This method attempts to minimize the sum of squared errors (SSEs) of any two nearby clusters that can be formed at each step. Conventionally, the clusters have been chosen from the distance matrix and from a threshold of distance taken on the dendrogram (red dashed line). The choice of a threshold is usually taken above small distances and where large jumps of distances can be observed (see Supplementary Fig. 8a,b). To assess the specificity of each cluster, we averaged the same *Z*-value used as an input to the algorithm over selected neurons (as shown in Supplementary Fig. 8c). Similarly, the firing rates of each neuron in the different clusters were averaged and displayed in Supplementary Fig. 8d. This revealed five clusters of single-unit activity in the MCC and three in the LPFC. This method emphasized that the strong representation of feedback (positive and negative) in the MCC was spread over different clusters (Clusters 3, 4 and 5). Importantly, a specific group of single units (cluster 4) contributed actively to both negative feedback and decision to Check (that is, bias towards positive *Z*-values for feedback and Check versus Work).

### Linear decoding methods

We applied multiple linear regressions to decode information from population activity vectors in both regions. This method assesses the capability of a linear readout to extract a given response variable (for example, Check versus Work) from trial by trial spike counts of the whole neuronal population. In this procedure, a Tikhonov regularization was used to minimize the SSEs and so to avoid overfitting by placing constraints on regression coefficients.

In this analysis, 60% of trials were used for training and 40% for testing, randomly selected from the pool of available trials. To determine the regularization parameter, we further subdivided the 60% partition to perform a five cross-validation procedure. For each regularization value tested, the SSEs across the five folds was calculated. The regularization value corresponding to the minimal SSE was then selected and used to train the classifier on the entire training partition (the 60% one). Finally, the testing was performed on the 40% partition, including only trials the classifier did not experience before. The classifier was trained and tested at each time bin.

To assess the temporal evolution of information coding, we applied this analysis at equivalent time points (by training and testing the classifier at a given time, see Fig. 5a) but also at different times (by training the classifier at time *t* and testing it at time *t+i*, see Fig. 5b). Such procedure highlights the dynamic of population coding, revealing the specificity (or generality) of pattern differences. Decoding matrices were thresholded to display only significant coding at *P*<0.05 (see below). For this analysis (performed for Feedback, Check versus Work and Cued decisions in the main task), spike counts were measured on successive bins of 200 ms for each 50 ms step and extracted from a set of 20 trials for each conditions. Owing to this under-sampling procedure, an unbiased readout performance (decoding rate) was extracted by randomly selecting trials and performing all computations 200 times. We then used the average decoding rate over these 200 computations. It is noteworthy that when comparing Check versus Work choices, we only considered trials that were preceded by a correct Cued decision in the main task, as monkeys mostly engaged in the Check option after correct trials (see Results).

A similar method was also used to assess how the population activity vector changed at different distances to Check (Fig. 8b,c), using a Lasso regularization. One advantage of the Lasso method is to reduce the number of predictors used (that is, neurons) depending on their importance and redundancy to discriminate the response variables (regression coefficient for unnecessary predictors are set to zero) and to allow feature extraction for instance. This allowed us to assess the minimal number of neurons that contributed significantly to the decoding. Venn representations on Fig. 8c show the number of neurons that had non-zero regression coefficient in at least 10% of the 200 pseudorandom trial selections. It is important to note that a high number of neurons with non-zero regression coefficient is not necessarily a guarantee of correct decoding. When the decoder could not fit the data (for example, did not converge), the decoding rate was set to 50%. For example, the decoder failed to converge for permuted data 49.7% of the time.

The same procedure as the one described earlier for the Tikhonov regularization was used for the Lasso regularization too, namely (1) the separation of trials into two independent partitions for training (60% of trials) and testing (the remaining 40%) purposes, and (2) the determination of the regularization parameter based on minimal variance during a fivefold cross-validation performed on the training partition. Here, the spike count was extracted from a set of 15 trials during the inter-trial period (from the EoT event to +2,000 ms) for each distance considered. We then compared the population activity for different distances of correct trials before a check (*n*, *n*−1, *n*−2 and *n*−3, with *n* being the trial at which the monkey checks) to a reference set of correct trials taken at a distance between five to ten trials before a check. This means that a correct decoding at trials *n*−1, for example, represents a significant difference in population activity between these trials and similar trials taken at *n*−5 to *n*−10, the only constant difference between the two groups of trials being the distance to the next check. Therefore, this approach allows to test whether traces of decision to check can be detected several trials before the actual choice of checking.

### Statistical procedures for linear decoding methods

We assessed statistical significance by performing 1,000 permutations of randomly shuffled conditions for each neuron. Trial assignments and all following computations were then performed using the same procedure as for the main tests (see above). Two-tailed *P*-values were then extracted by counting the number of permuted observations that have a greater value than actual observation, divided by the number of permutations. We used a cutoff threshold of 95%.

In addition, statistical comparisons between MCC and LPFC population coding were performed using KW tests (with Bonferroni correction for multiple testing) over the 200 random trial selections. Only time bins where at least one of the two populations significantly decoded the information were tested and reported on Fig. 5.

### Comparison of linear and nonlinear classifiers

It must be noted that numerous algorithms for classifications have been developed in the field of machine learning and statistics; all of them could in principle be used to assess how neuronal population activities discriminate response variables (foe example, see ref. 28). The linear decoding methods we performed here (see Figs 5 and 8b,c) represent only one type of algorithm, which tends to be preferred in electrophysiology studies given their few advantages. In particular, they do not rely on complex relations between predictors and response variables; they only use a hyperplane to separate the input data. This means that only one downstream neuron could theoretically read out and discriminate response variables by simply performing a linear sum of the inputs from a whole population. In addition, such methods are less computationally demanding than nonlinear versions and can therefore be performed easily, yet providing accurate predictions. Nevertheless, it is possible that linear methods did not provide the best decoding accuracy in some contexts (for example, underestimated accuracy in case of a nonlinear encoding of the response variables), or even tend to overestimate possible effects.

To test the reliability of our results for the different conditions considered, we also applied an alternative nonlinear classifier, using support vector machine (SVM) with Gaussian kernel. Contrary to linear classifiers, SVM mapped nonlinearly input data into a higher-dimensional space and separated conditions by using non-planar boundaries. In the case of a nonlinear encoding of the response variables, we might expect more accurate predictions than previously observed with a linear separation. Alternatively, a linear coding will bring similar results. To ensure a fair comparison, all procedures (trial selection, assignments into training/testing sets and permutations) were conducted similarly to the linear decoding (see above).

Average decoding accuracies for both linear classifier (from Fig. 5) and nonlinear SVM are shown in Supplementary Fig. 7. Striking similarities could be noted between both classifiers types, for all three response variables (Feedback, Check versus Work decision and Cued decisions in the main task). No differences could be observed in terms of strengths or latencies of significant decoding, suggesting that simpler linear boundaries are enough to account for the coding of these variables in MCC and LPFC populations.

### Data availability

All relevant data and codes are available from the authors upon request.

## Additional information

**How to cite this article:** Stoll, F. M. *et al.* Specific frontal neural dynamics contribute to decisions to check. *Nat. Commun.* 7:11990 doi: 10.1038/ncomms11990 (2016).

## Supplementary Material

Supplementary Information Supplementary Figures 1-12
