# Supplementary material for: Specific frontal neural dynamics contribute to decisions to check
Source: Nat Commun. 2016 Jun 20;7:11990. doi: 10.1038/ncomms11990 (PMC4915137; doi:10.1038/ncomms11990)
Supplement: Supplementary Information — Supplementary Figures 1-12 [file ncomms11990-s1.pdf]

Supplementary Information

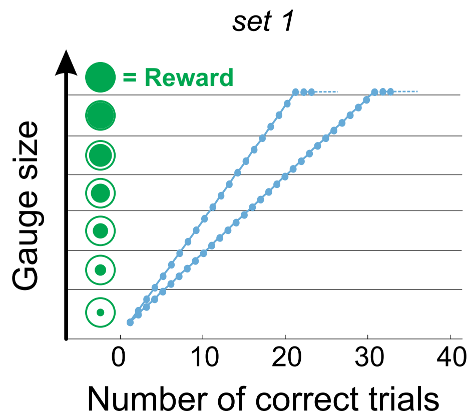

Supplementary Fig. 1. Principle of the Gauge increase. The gauge size (*y* axis) increases linearly following 7 steps depending on the number of correct Cued trials (*x* axis) performed in the main task. The total number of correct trials required to earn each bonus varies randomly from one block to another, defining different speed of gauge increase. It was 21 or 31 trials in a first set of sessions (left, used for monkey D) or 14, 21, 29 or 36 trials in a second set (right, used during the recording period for monkeys A and H). Note that the gauge stays full until monkeys check for it (dashed blue lines on top).

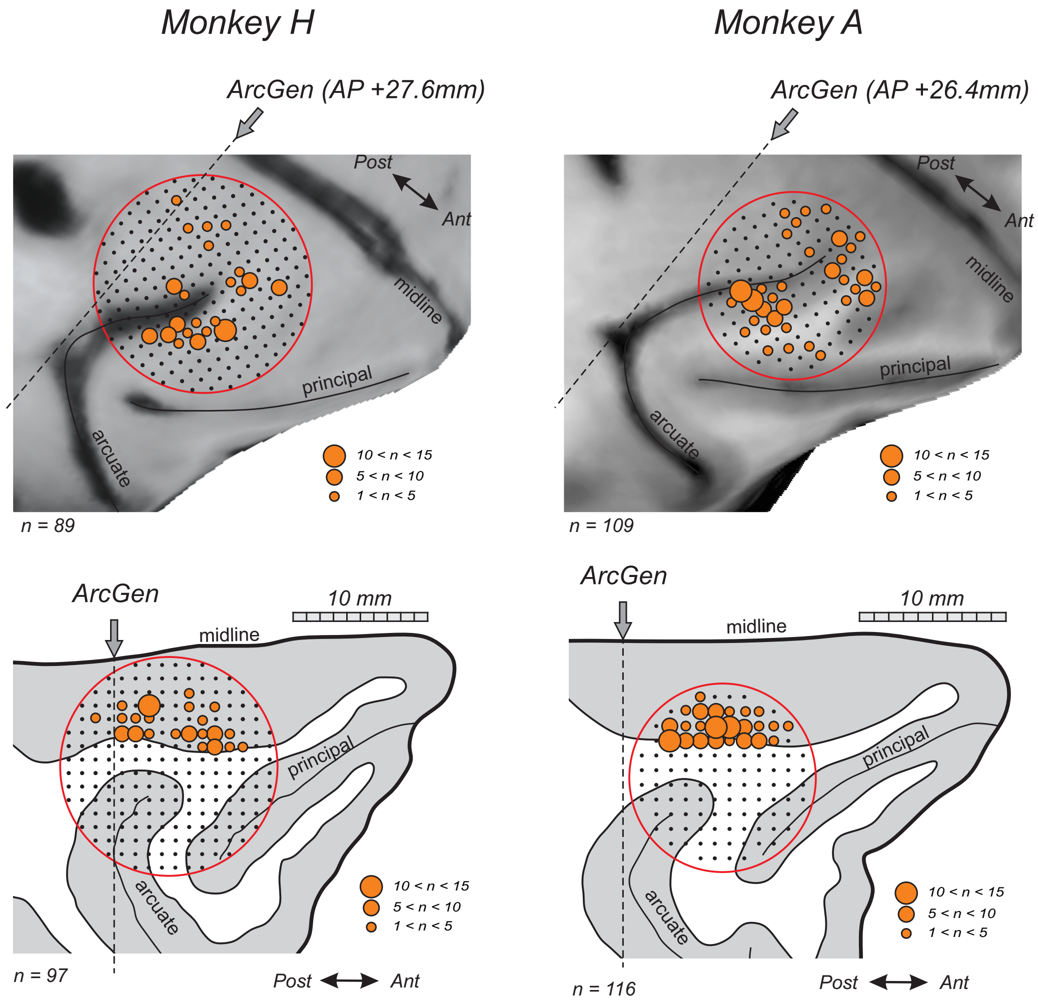

Supplementary Fig. 2. Recording locations in monkey H and A. Recording grid reconstructions from neuronavigation and from each monkey's anatomical MRI (T1, 1.5T). **Top,** reconstructions of a grid over the 3D surface of the cortex to show the location of recordings in the dorsal frontal and prefrontal cortex (LPFC). Major sulci are indicated, as well as the rostro-caudal level of the genu of the arcuate sulcus (Arc Gen). **Bottom,** line drawing of the cortex of the dorsal bank of the cingulate sulcus (MCC) taken from an MRI section perpendicular to the electrode tracks. Colored disk size indicates the number of single units recorded at each location. *'n'* indicates the total number of single units for each structure in each monkey.

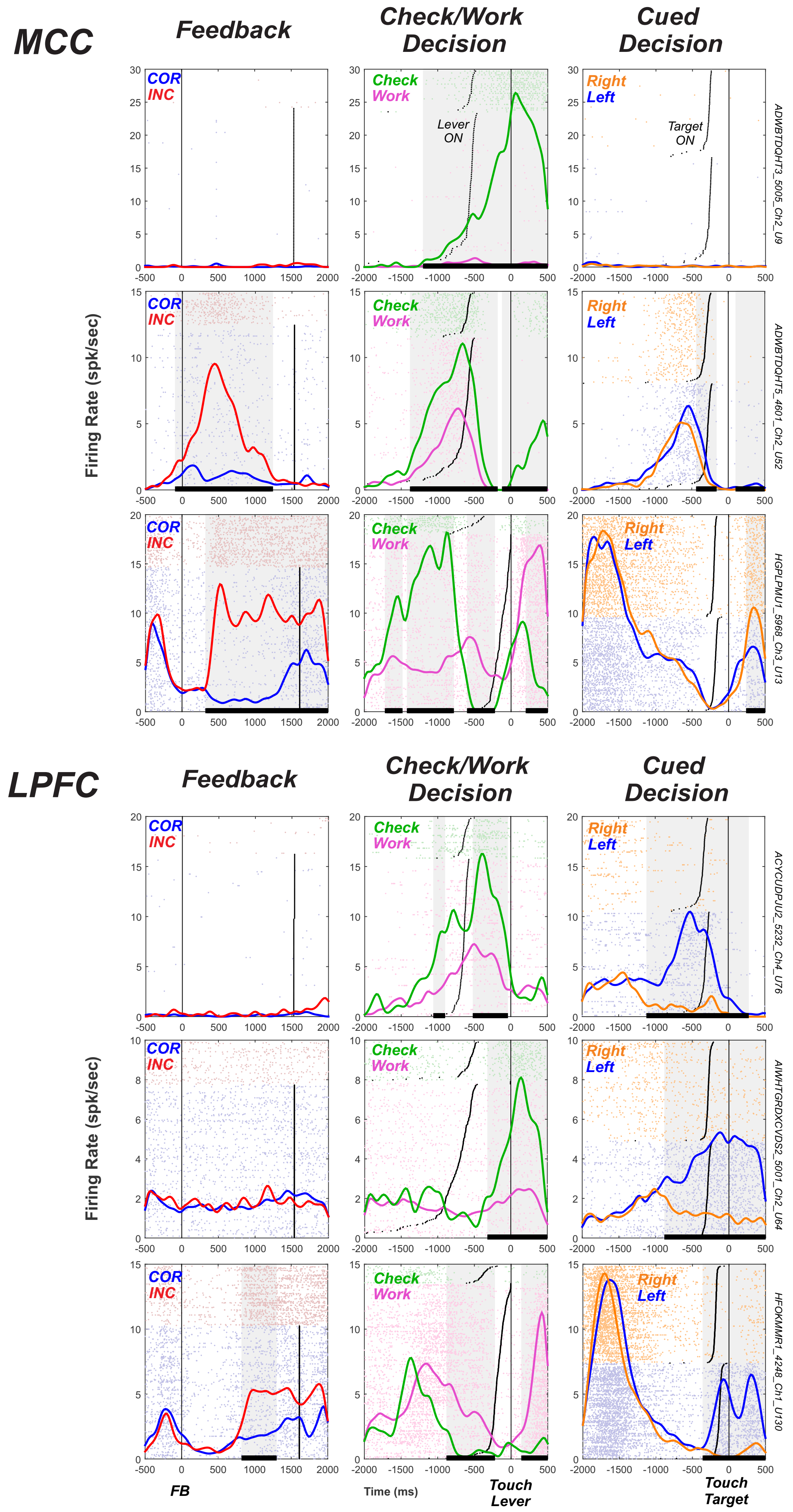

Supplementary Fig 3. Examples of single unit activity. Average firing rates and rasters for 6 different single unit activities in MCC (top 3 rows) and LPFC (bottom 3 rows) during negative/positive feedback (Left), Check vs. Work decisions (Middle), and Cued decisions in the main task (Right). Black lines on time axis and gray background highlight the time periods where the difference in firing rate is significant (Kruskall-Wallis test, p<0.01).

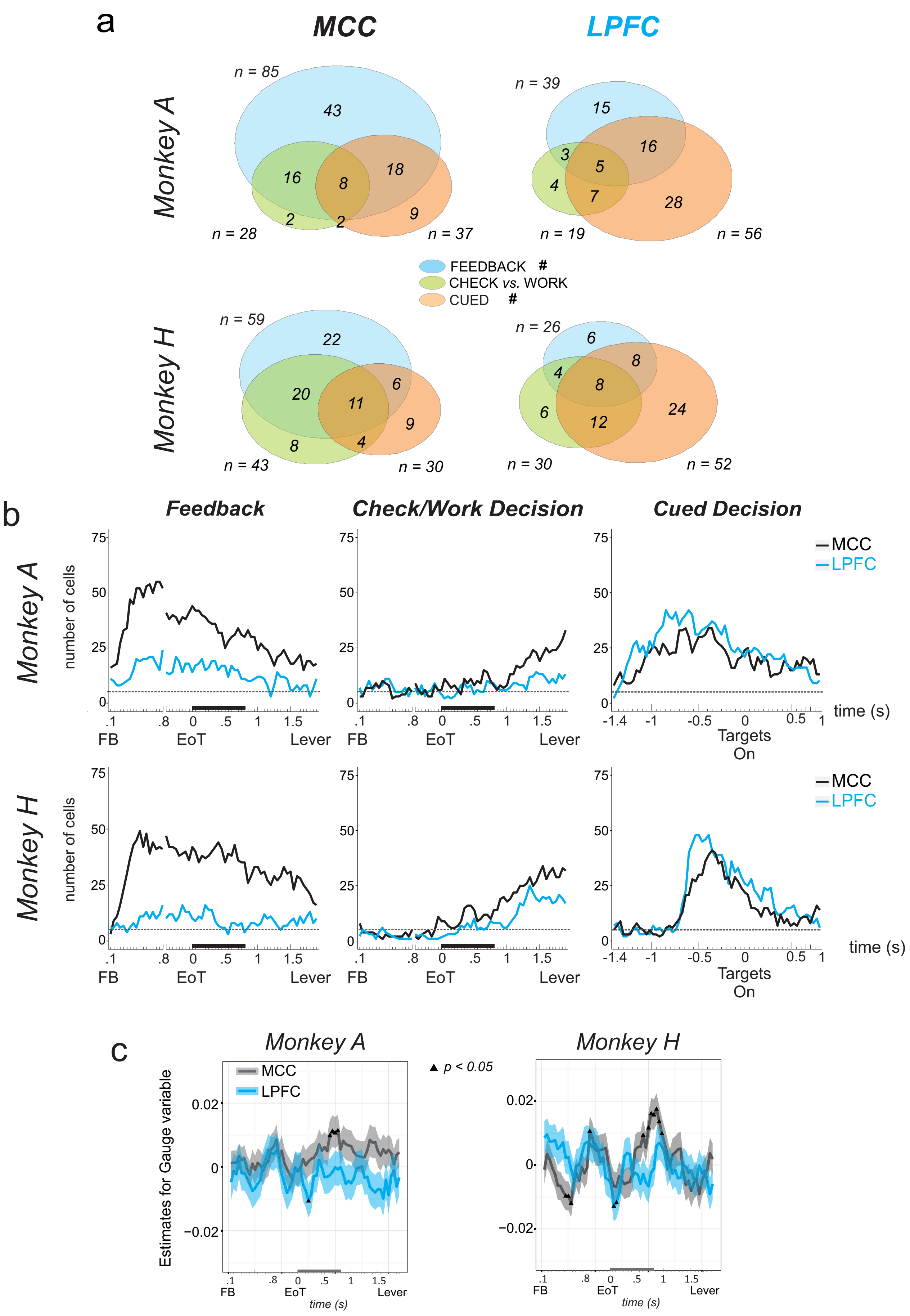

Supplementary Fig. 4. Single-subject data. The figures present population data for each monkey (Monkey A and H) with conventions as in main **Figs. 4** and **7a**. (**a**) Population data of neurons presenting discrimination for Feedback, Check *vs.* Work decisions, and Cued decisions in the main task. Symbol # indicates a significant difference between regions for both monkeys (Chi-square tests, *p*<10-2). (**b**) Sliding *glm* on single unit activity for Feedback, Check *vs.* Work, and Cued decisions in the main task. (**c**) Estimates for the covariate Gauge extracted from the population *glm* (see main text and Methods).

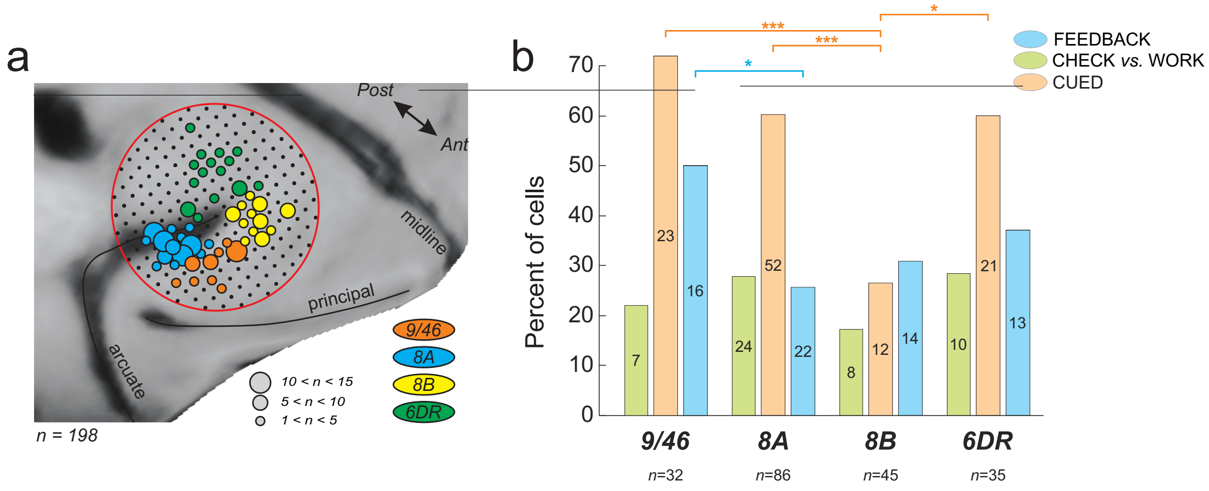

Supplementary Fig. 5. Homogeneous coding between regions identified in the LPFC. (**a**) Recording grid reconstruction pooled for both monkeys and highlighting the number of recorded neurons in each LPFC subregions. The separation between areas is based on the description made in Morecraft et al. *Cytoarchitecture and cortical connections of the anterior cingulate and adjacent somatomotor fields in the rhesus monkey*. Brain Res Bull 87, 457-497 (2012). (**b**) Proportions of neurons (and the actual number within bars) discriminating Feedback, Check *vs.* Work and Cued decisions in each area. Significant Chi-square comparisons are reported on the figure: * $p<0.05$, *** $p<0.001$. The main difference was a smaller discrimination of Cued decisions in area 8B compared to the others. However the number of recorded neurons in each area was not sufficient to go further in the differentiation between areas.

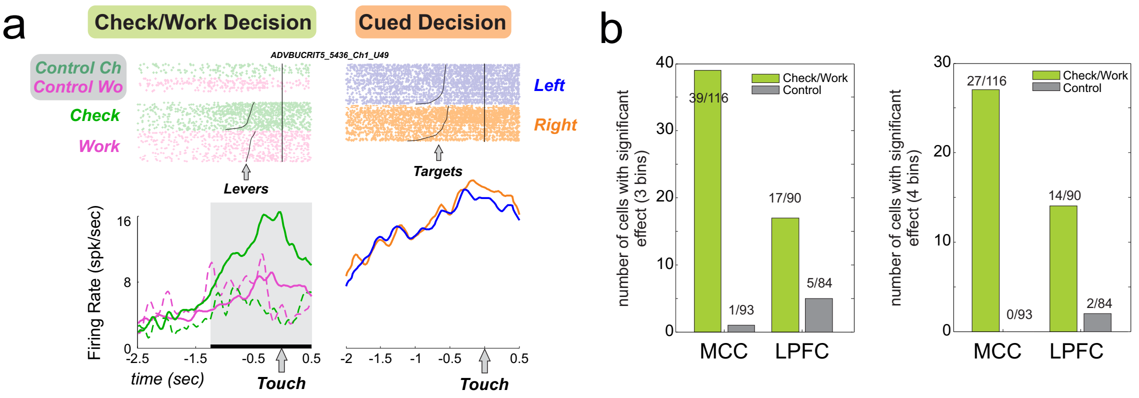

Supplementary Fig. 6. Check vs. Work compared with the Control task. The control task consists in touching a visual item presented at the position of one of the two levers. No reward is given (see Method). (**a**) One typical example neuron (recorded in the MCC) discriminating Check *vs.* Work decisions but not control trials (left panel, green and pink dashed lines, and rasters on top) nor Right *vs.* Left choices in cued decisions (right panel) (green: Check, pink: Work, Blue: Left, Orange: Right choices). (**b**) Number of cells in each structure for which a significant effect of Check *vs.* Work (green) or of Control Left *vs.* Right (grey) were observed. The two histograms present the data for 2 different thresholds, 4 successive significant time bins (on the right, as in the main data analyses), and at a weaker threshold (3 bins, on the left). The figure shows that even at low threshold single units were very much involved in encoding Check *vs.* Work but not the spatial position of the lever in the Control task.

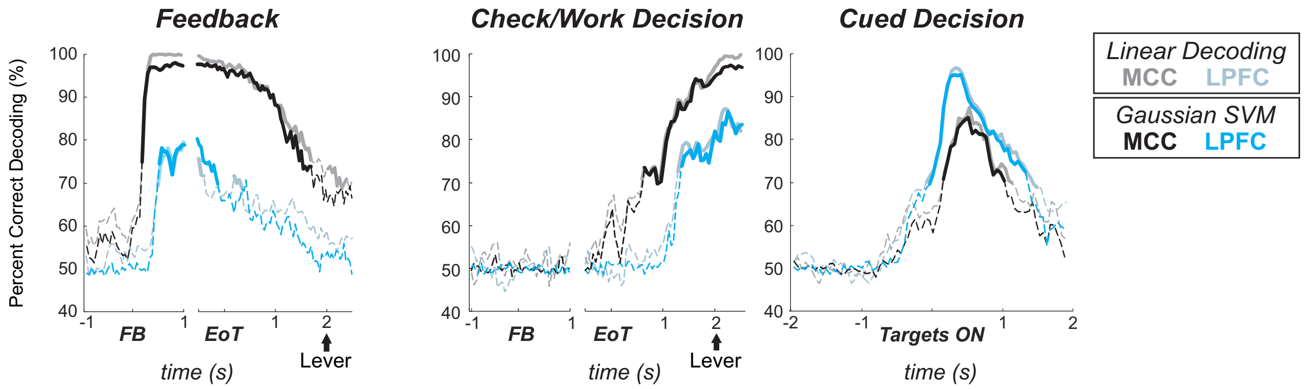

Supplementary Fig. 7. Comparison of population coding using linear and non-linear decoding methods. The results obtained from non-linear support vector machine (SVM) classifiers with a Gaussian kernel (bright black and blue, for MCC and LPFC respectively) are superimposed on the results shown in **Fig. 5a** using linear decoding methods (light colors). Both methods used the same overall procedures (see Method). Only the 200 random trial selections differ between classifiers, which could in principle account for minor deviations. Nevertheless, non-linear SVM classifiers perform as well as linear classifiers, both in terms of strength or latency of significant decoding (highlighted in bold, $p<0.05$).

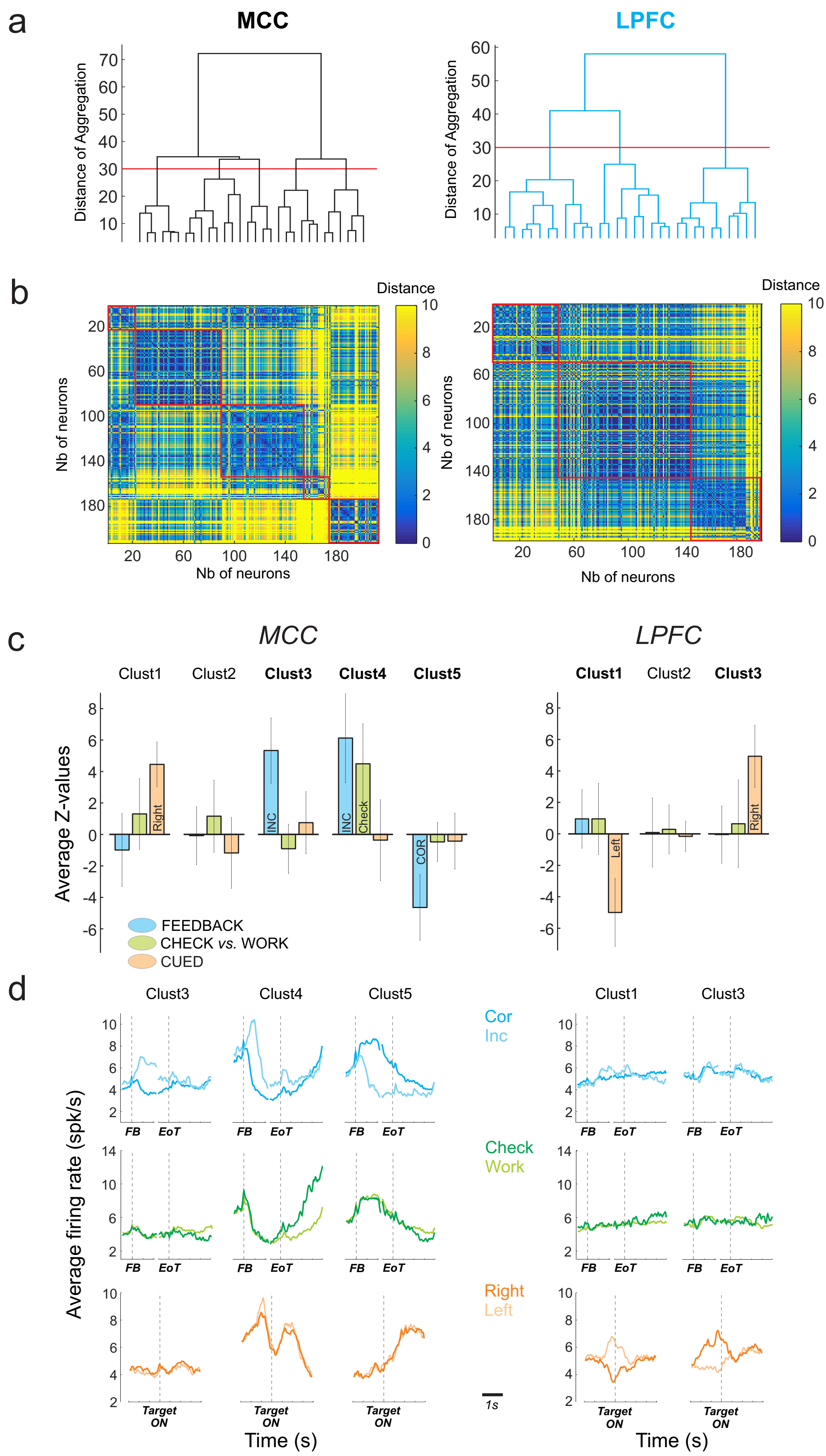

Supplementary Fig. 8. Hierarchical clustering of SUAs. (**a**) Dendrograms for MCC (left) and LPFC (right) representing the trees of classification constructed by the hierarchical clustering algorithm. (**b**) Similarity matrices obtained from the dendrogram at a specific threshold (red line in **a**). Neurons are sorted based on their appartenance to a given cluster. (**c**) Average z-values of each detected clusters for the 3 parameters considered (Feedback, Check *vs.* Work and Cued decisions) revealing classes of neurons with different encoding properties. The sign of z-values indicates the selectivity indicated within bars (e.g. positive z-values for feedback indicate more activity for Negative (INC) than for positive feedback). (**d**) Average firing rate around feedback and EoT (top and middle rows) and targets selection (bottom row) for the main clusters displayed in **c** (Clusters 3, 4 & 5 for MCC, and 1 & 3 for LPFC, from left to right). The clustering procedure highlights the preponderance of feedback in the MCC as well as the co-expression of feedback and check-related activity. However, LPFC neurons were mostly grouped depending on their Cued decision preference in the main task.

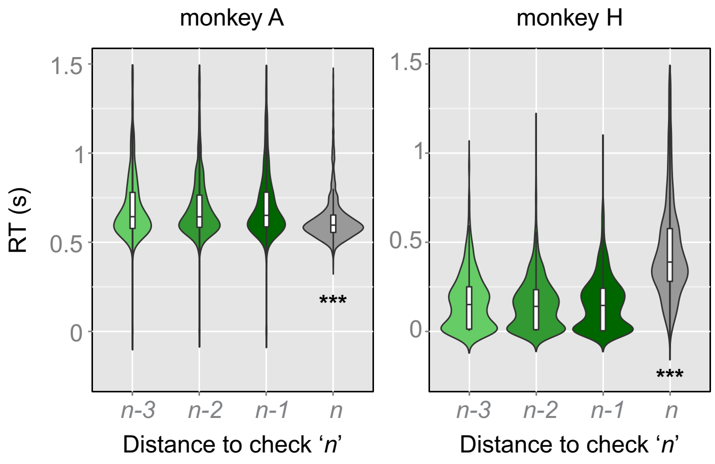

Supplementary Fig. 9. Check/Work response times. Violin representations of Check and Work response times depending on the distance to check, at trial n, for monkey A (left panel) and H (right panel). Distributions of response times were compared using a two-sample Kolmogorov-Smirnov test, revealing a significant differences between trial n compared to all others (*n-1*, *n-2* and *n-3*; *** *p*<10-30). Other comparisons (between *n-1*,*n-2* or *n-3*) were not significant (*p*>0.35). Hence, the figure shows that monkeys' response times differed between Check and Work decisions but did not vary with the distance to check (*n-3* to *n-1*, i.e. between Work decisions).

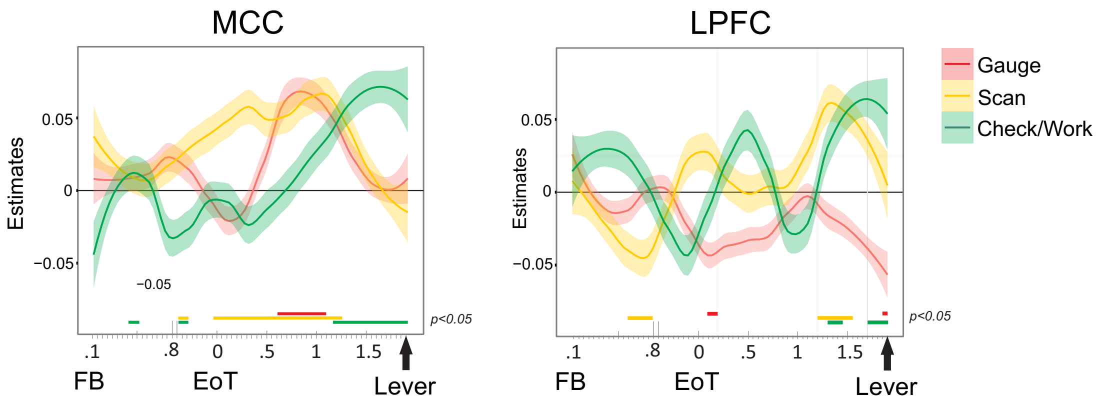

Supplementary Fig. 10. Fixed effects of Scan, Gauge and Check/Work decision on group activity in MCC (left) and LPFC (right). The estimates correspond to the isolated fixed effects from the *glmm* (see Methods). We tested and found that the interaction bin-per-bin between Scan and Check/Work was not significant. Nevertheless, both have a positive influence on group activity in MCC: activity is higher when the monkey checks, and when he scans both levers. The horizontal lines indicate which bins (equal or more than 2 successive) are at p<0.05.

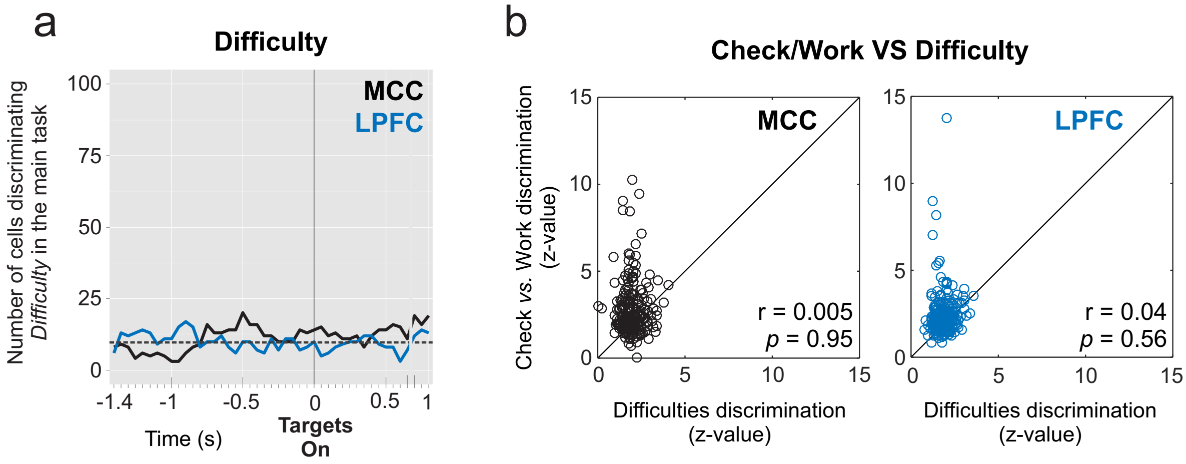

Supplementary Fig. 11. Number of neurons encoding difficulty in the main task and its relation with encoding Check vs. Work. (a) Time resolved number of cells extracted from the sliding *glm* with a significant discrimination of difficulty in the main task**.** Dashed gray line represents the 5% level. **(b)** Scatter plot showing the relationship between absolute maximal z-values for Check vs. Work discrimination for each neuron (extracted from the sliding *glm,* y-axis) and difficulty at the time of target onset in the main task (x-axis). Note that only weak, if any, encoding of difficulty was observed, and note also the absence of correlation between Check and Difficulty effects in both areas (r and p indicated within the panels).

.

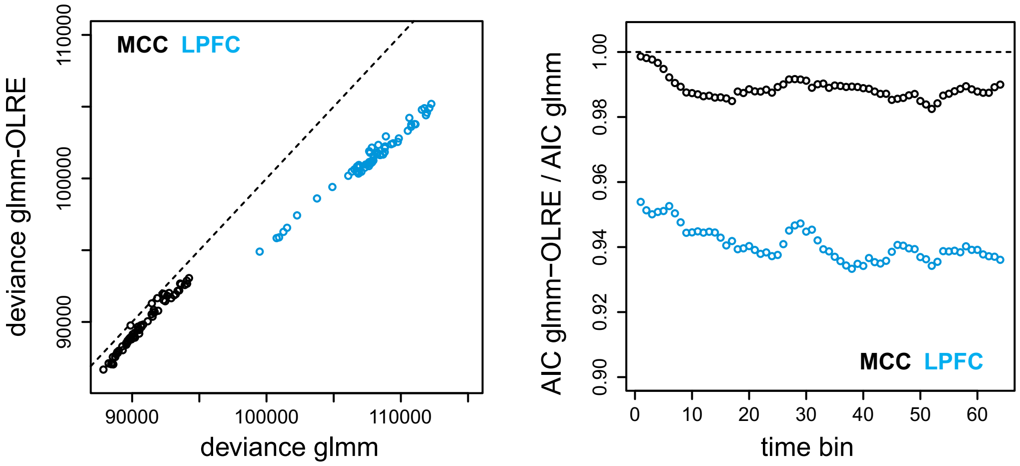

Supplementary Fig. 12. Model comparisons between poisson *glmm* with or without the Observation-Level random effects (OLRE). The models named glmm and glmm-OLRE are compared for deviance (right) and AIC (left: ratio of the two AIC across time bins (for the two structures, MCC and LPFC). The models included Gauge, CheckWork, and PreviousPerformance as fixed effects and SUA as random effect. Note that the glmm-OLRE has always smaller deviance and smaller AIC.
